# Therapeutic Potential of Anesthesiology for Sleep Disorders in the Perioperative Period

**DOI:** 10.2174/011570159X368375250611103614

**Published:** 2025-06-30

**Authors:** Xiao-li Pan, Yu-fan Xi, Peng Liang, Jiapeng Huang

**Affiliations:** 1 Department of Anesthesiology, West China Hospital, Sichuan University, Chengdu, Sichuan Province, 610041, China;; 2 West China School of Medicine, Sichuan University, Chengdu, Sichuan Province, 610041, China;; 3 Day Surgery Center, General Practice Medical Center, West China Hospital, Sichuan University, Chengdu, Sichuan Province, 610041, China;; 4 Department of Anesthesiology and Perioperative Medicine, University of Louisville, Louisville, KY, 40202, United States

**Keywords:** Anesthesia, sleep, insomnia, sleep apnea, dexmedetomidine, propofol

## Abstract

Sleep is important to maintain normal physiological functions of the human body. With increased stress in modern society, the number of patients suffering from sleep disorders is gradually increasing. Many studies have shown that general anesthetics induce loss of consciousness by acting on the sleep-wake circuit. In recent years, general anesthesia and other anesthetic agents have been used in the diagnosis and treatment of sleep disorders. This article discusses the mechanism of sleep and sleep disorders, summarizes the effects of anesthetics on sleep and their regulatory mechanisms, and reviews the research progress of using anesthetics in the diagnosis and treatment of sleep disorders.

## 1 INTRODUCTION

In humans and animals, general anesthesia induces a condition of reduced responsiveness that anesthetists and patients refer to as ‘sleep’ [1]. Despite the behavioral similarities between anesthesia and sleep, the two states can be distinguished by their unique physiological and behavioral features [2]. Unlike sleep, general anesthesia does not happen spontaneously, cannot be reversed by outside stimuli, and is typified by an electroencephalogram (EEG) pattern that lacks the discrete stages and the cycling between stages that characterize sleep in the natural world. Furthermore, sleep is controlled both homeostatically and by circadian cues, and it can be disrupted by external influences. In contrast, the duration and depth of general anesthesia are essentially determined by the agent dose and the period of administration.

The behavioral parallels between anesthesia and normally occurring sleep have shown that the states share underlying neurophysiological similarities. Recent research on the effects of anesthesia on specific brain nuclei and molecular receptors has revealed that the alterations in brain activity brought on by anesthesia administration closely resemble those that take place during sleep [3]. Additionally, the tuberomammillary nucleus (TMN), a recognized brain region involved in sleep regulation, appears to be the site of certain receptor-based effects of general anesthetics [4]. All of these results suggest that the effects of anesthetics on the neural networks that are typically engaged in producing or sustaining naturally occurring sleep may contribute to some of the features of general anesthesia. Animal behavioral research has suggested that anesthesia and sleep may have similar neurophysiological components, indicating that both states may produce similar effects. Research on rats indicates that recovery from sleep deprivation can happen under anesthesia and that sleep needs do not increase during extended anesthesia [5].

**Fig. (1):**
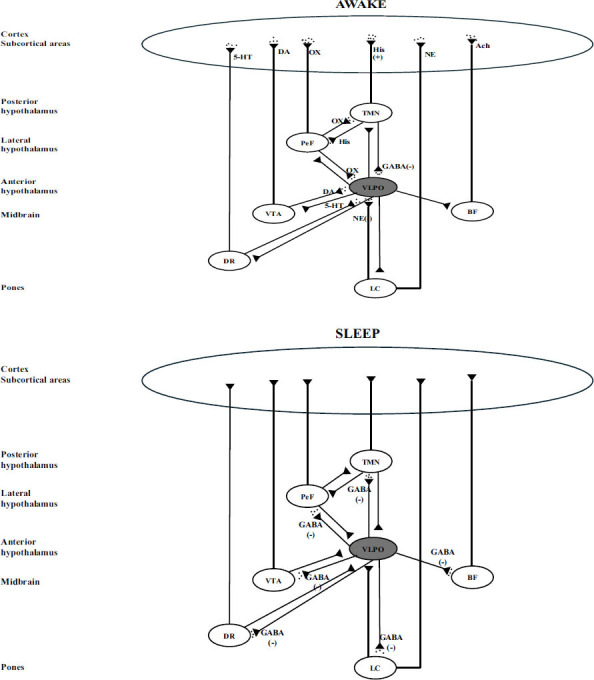
Working model of intrahypothalamic sites involved in sleep regulation and the pharmacological and anatomic interactions between these sites. **Abbreviations**: TMN: Tuberomammillary Nucleus, VLPO: Ventrolateral Preoptic Nucleus, LC: Locus Coeruleus, BF: Basal Forebrain, PeF: Perifornicular Region, VTA: Ventral Tegmental Area, DR: Dorsal Raphe.

In recent years, with the development of anesthesia therapy and sleep medicine, general anesthetic drugs and anesthesia techniques have been studied and applied in the diagnosis and treatment of sleep disorders. Dexmedetomidine, a highly selective alpha-2 adrenergic receptor agonist, has been used in the diagnosis and treatment of sleep disorders using its effects in inducing a sleep-like state, minimal respiratory depression, and neuroprotection [6]. Propofol is the most widely used anesthetic for general anesthesia. Research has indicated that propofol can enhance the quality of sleep for people undergoing surgery and intensive care unit stays [7, 8]. Stellate ganglion block has also been demonstrated to improve postoperative sleep quality (Fig. **1**) [9].

In this review, the mechanism of sleep and sleep disorders is discussed, the effect of anesthetics on sleep and their regulatory mechanisms is summarized, and the research progress of using anesthetics in the diagnosis and treatment of sleep disorders is reviewed.

## 2 METHODS

A systematic review approach was utilized to provide a comprehensive overview of existing research evidence about the therapeutic effect of anesthetics on sleep disorders. A comprehensive search strategy was conducted in the PubMed database from its inception till the 27^th^ of December 2024.

The specific search strategy employed was as follows:

(“Anesthetics” [MeSH] OR “Anesthetics, Intravenous” [MeSH] OR “Propofol” [MeSH] OR “Ketamine” [MeSH] OR “Dexmedetomidine” [MeSH] OR anesthe* [tiab] OR sedative* [tiab] OR “intravenous anesthetic*” [tiab]) AND (“Sleep Wake Disorders” [MeSH] OR “Sleep Initiation and Maintenance Disorders” [MeSH] OR “Disorders of Excessive Somnolence” [MeSH] OR “REM Sleep Behavior Disorder” [MeSH] OR insomnia [tiab] OR hypersomnia [tiab] OR “sleep disorder*” [tiab] OR “sleep quality” [tiab] OR “sleep improvement” [tiab]) and the total yield was 3711 papers. The reference lists of relevant primary papers and reviews were also reviewed to identify studies that may have been missed in the search. Finally, 46 papers were adopted. Because each section of the article has duplicate content, the specific number of papers cannot be determined for each section.

## 3 SLEEP AND SLEEP DISORDERS

Sleep is regulated by specific brain regions, as well as their accompanying neurons and nerve fibers. The endogenous sleep-wake circuit and its related endogenous chemicals primarily regulate sleep. Franks *et al.* [10] defined sleep as “a naturally occurring, periodic state of rest during which consciousness of one’s environment and responses to external stimuli are largely suspended”. Sleep can be classified into two stages based on EEG changes: rapid eye movement (REM) sleep and non-rapid eye movement (NREM) sleep. Depending on the depth of sleep, NREM can be further classified into N1, N2, and N3 stages [11]. The wake-promoting nuclei, which have arousal function and are active during wakefulness, mainly include the Locus Coeruleus (LC), Tuberomammillary Nucleus (TMN), and Basal Forebrain (BF). Sleep-promoting nuclei are active during sleep, mainly the ventrolateral preoptic nucleus (VLPO) [12]. When entering the sleep state, the excitability of VLPO neurons increases significantly, and gamma-aminobutyric acid (GABA) is released, which inhibits the activities of TMN and LC and blocks their excitatory effects on the cortex.

Sleep disorders are defined as poor sleep quality and aberrant sleep behavior, which are manifestations of disturbances in sleep and waking cycles. According to the revised version of the International Classification of Sleep Disorders, 3^rd^ edition (ICSD-3-TR) [13], sleep disorders are classified into seven major categories, including insomnia disorders, sleep-related breathing disorders, central disorders of hypersomnolence, circadian rhythm sleep-wake disorders, sleep-related movement disorders, parasomnias, and other sleep disorders [14]. Lack of sleep can have a detrimental effect on physiological function, hospitalization, satisfaction, and patient prognosis. Physicians must diagnose sleep disturbances as soon as possible and help patients as needed [15]. Sleep disturbances in patients undergoing surgery have a complex, multifaceted impact on depression, weariness, and pain. The following are some adverse effects that sleep disorders have on patients' outcomes.

There is an interaction between sleep and pain. In previous studies, individuals with breast cancer who slept poorly before surgery experienced more pain after the surgery [16]. A strong correlation was found between the degree of pre-operative insomnia and post-operative pain. Post-operative discomfort was the primary cause of disrupted sleep in about 48% of patients on the first postoperative day [17]. Additionally, patient satisfaction was favorably correlated with pre-operative sleep efficiency [18]. This may be related to transient sleep disruptions that exacerbate hypersensitivity to pain following surgery. In a pilot study of patients having joint replacements, an increase in preoperative sleep duration decreased pain and opioid use in the first three to four days after surgery [19]. Although most of the current literature supports perioperative sleep disturbance as a risk factor for post-operative pain, the precise processes are not entirely understood. It is hypothesized that short-term perioperative sleep deprivation may raise the lumbar dorsal root ganglion's L-type calcium channel expression and activity, which would delay the recovery from post-operative pain [20].

Additionally, insomnia and depression are related; individuals with depression have high rates of insomnia and sleep disturbances, and insomnia is a powerful predictor of depression development. A higher proportion of post-operative mental health disorders is typically associated with a greater percentage of sleep problems. After bariatric surgery, people who have sleep disturbances may do non-fatal self-harm or commit suicide [21]. Sleep disruptions are a clinical sign of mental disorders as well as a risk factor. Prior studies suggested a possible link between pre-existing sleep disorders and postoperative delirium [22]. According to a study on heart surgery, pre-operative sleep disturbance was the primary predictor of postoperative delirium [23], particularly in older adults [24]. Compared to older persons without sleep disorders, those who experienced chronic sleep disturbance prior to hospitalization had a considerably increased incidence of postoperative delirium. A correlation was found between insomnia and negative cognitive development. According to Ni *et al*. [25], preoperative sleep disorders may worsen postoperative cognitive impairment in older mice by aggravating surgically induced neuroinflammation, nerve injury, and blood-brain barrier disruption.

## 4 EFFECTS OF ANESTHETICS ON SLEEP

There are two different ways that general anesthetics affect sleep. To some extent, general anesthetics can promote sleep. General anesthetics can efficiently restore sleep behaviors in sleep-deprived rats, producing similar effects as natural sleep [26]. Zhang *et al.* [27] shown that multimodal hypnosis utilizing sedative medications, including dexmedetomidine, propofol, and ketamine, in conjunction with other treatment modalities has a considerable therapeutic effect on intractable insomnia. However, general anesthetics can also disrupt sleep. Su *et al.* [28] discovered that sevoflurane may increase the occurrence of postoperative sleep disturbances in surgical patients, resulting in the disruption of sleep rhythms, reduction in total sleep time, and decreased sleep quality.

General anesthetics can cause sedation and hypnosis by inhibiting wake-promoting nuclei (LC, BF) or activating sleep-promoting nuclei (VLPO) in the brain [29]. The mechanism of general anesthetics on sleep mainly comprises three aspects: (1) altering sleep structure [30], (2) influencing circadian rhythms, and (3) regulating the sleep-wake loop.

### 4.1 Altering Sleep Structure

Intravenous anesthetics can alter the structure of sleep and have varying degrees of impact on sleep. Tung *et al*. [26] conducted electrophysiological sleep-wake recordings in rats sedated with propofol 297 ± 38 μg·kg^-1^·min^-1^ for 6 hours after 24 hours of sleep deprivation. There was no rebound increase in REM and NREM sleep after propofol infusion, indicating that anesthesia also causes a recovery process similar to that which happens during naturally occurring sleep. Dexmedetomidine is a potent α2-adrenoceptor agonist that primarily targets the locus coeruleus in the brain. Akeju *et al*. [30] administered dexmedetomidine to healthy participants and monitored them with polysomnography. There was no significant change in total sleep time in dexmedetomidine-administered healthy volunteers, but the NREM time was extended by 33.2 minutes compared to natural sleepers, without significant differences in N1 and N2 sleep times. The duration of N3 sleep increased by 35.8 minutes, which was compensated by REM sleep, implying that dexmedetomidine can promote the extension of NREM stage 3 sleep. Furthermore, oral administration of dexmedetomidine was found to decrease the duration of REM sleep, extend the duration of NREM sleep, and shorten the waking time [30, 31]. Dexmedetomidine may help to stabilize sleep and enhance sleep quality by altering the sleep structure.

Inhaled anesthetics can also affect sleep architecture. Zhang *et al*. [32] demonstrated that sevoflurane and isoflurane affected sleep architecture in the mouse brain by interacting with G-protein signaling proteins and Galphai2, resulting in shorter awake and longer NREM and REM sleep. Jia *et al*. [33] demonstrated that REM sleep duration was prolonged, and the frequency of REM sleep episodes increased in elderly mice following 2 hours of sevoflurane exposure, suggesting that inhaled anesthetics may disrupt sleep structure and contribute to sleep disorders.

The beneficial effects of general anesthetics on sleep structure can help to stabilize sleep, enhance sleep rhythm, ease insomnia symptoms, improve sleep quality, and effectively treat sleep disorders. The disruptive effects of general anesthetics on sleep structure could be minimized by reducing anesthetic dosages and implementing individualized programs for surgical patients to reduce the incidence of postoperative sleep disorders and improve patient prognosis.

### 4.2 Influencing Circadian Rhythms

The term “circadian rhythm” is the regular change of life activities for a 24-hour period. A master clock located in the hypothalamic suprachiasmatic nucleus mostly regulates the central and peripheral clocks that comprise the circadian rhythm system. At the molecular level, the main components of the circadian clocks include BMAL1, CLOCK, PERIOD (PER, including PER1, PER2, and PER3), and CRYPTOCHROME (CRY, including CRY1 and CRY2) [34]. The circadian rhythm regulates sleep rhythm, and disruptions to this cycle can cause sleep problems in different degrees.

General anesthetics can disrupt the circadian clock and change the circadian rhythm by affecting the expression of clock genes and regulatory proteins. Dunlap *et al*. [34] demonstrated that general anesthesia can directly impact the expression of core clock molecular genes in the hypothalamic suprachiasmatic nucleus (SCN), resulting in phase delay, altered circadian rhythm, and sleep problems. PER2 functions as a negative regulator of the feedback regulation pathway. Yoshida *et al*. [35] discovered that dexmedetomidine and propofol suppressed the production of PER2, resulting in a delayed circadian phase and altered circadian rhythm. According to Matsuo *et al*. [36], sevoflurane also suppressed PER2 expression in the hypothalamic suprachiasmatic nucleus. Therefore, general anesthetic agents may directly or indirectly alter the expression of core clock molecular genes in the SCN, causing phase alterations in circadian rhythm and contributing to sleep rhythm disorder.

Melatonin is a neuroendocrine hormone that regulates the sleep-wake cycle and circadian rhythm [37]. Melatonin can help with sleep disorders and restore normal sleep. Melatonin secretion disturbances can affect circadian rhythms. Dispersyn *et al*. [38] found that intraperitoneal injection of propofol 10 mg/ml in rats disrupted the rhythm of melatonin secretion, as evidenced by a significant decrease in melatonin secretion 3 hours after anesthesia and a significant increase in melatonin secretion 20 hours later. After 4% isoflurane exposure, rats' blood melatonin concentrations were considerably lowered, and their circadian rhythm was significantly disrupted.

General anesthetics can disrupt circadian rhythms through multiple mechanisms at different levels, resulting in sleep disorders. As a result, limiting the influence of general anesthetics on circadian rhythm may lower the prevalence of sleep disorders.

### 4.3 Regulating the Sleep-Wake Loop

The sleep-wake loop is an important mechanism to regulate the sleep-wake cycle. There are important nuclei in the sleep-wake loop in different brain regions, which play an important role in the normal sleep-wake function. General anesthetics could induce loss of consciousness by acting on the sleep-wake-related brain regions.

Ventral Tegmental Area (VTA) dopaminergic neurons have a significant role in promoting wakefulness *in vivo*. Increased dopamine production suppresses sleep. Qian *et al*. investigated the loss of righting reflex, recovery of righting reflex, and sleep duration of rats under propofol and isoflurane anesthesia in the lesion group (bilateral VTA was given the specific dopamine neuron damage drug 6-hydroxydopamine) and the control group (bilateral VTA was given the same volume of normal saline). They revealed that, compared to the control group, rats in the lesion group slept substantially longer following propofol and isoflurane anesthesia, which could be attributed to a decrease in dopaminergic neurons in the VTA. These findings indicate that anesthetics may influence sleep by suppressing dopaminergic neurons in the ventral tegmental region of the midbrain. Orexin neurons in Pef neurons are required for physiological sleep, and disrupting this system is thought to be a primary cause of sleep disorders and narcolepsy [39]. Zecharia *et al*. [40] studied GABAA receptor β3-N265M knock-in mice using an electrophysiological patch-clamp technique and found that propofol inhibited orexin neurons in the Pef area to varying degrees. Propofol may influence sleep by suppressing the release of orexin in Pef neurons.

## 5 RESULTS OF CLINICAL EVIDENCE

### 5.1 Chronic Insomnia

A total of 7 studies were included in this review to explore the therapeutic effect of anesthetic drugs on insomnia. There were 4 clinical studies and 2 case series on dexmedetomidine in the treatment of insomnia and 1 clinical study on the benefit of propofol in the treatment of refractory insomnia.

Two case reports were designed to describe the use of dexmedetomidine at home in children with insomnia, 9 *via* intranasal, and 1 *via* intravenous route [41, 42]. At home, dexmedetomidine was administered for 3000 days (minimum 1 month, maximum 36 months). All patients reported a long-lasting improvement in their symptoms, and none of them discontinued dexmedetomidine due to side effects or perceived lack of therapeutic efficacy. Although the use of propofol at home shows promise in treating intractable sleep disturbances in pediatric palliative care children, more research is required to validate these findings. Based on the ideals of Patient-Controlled Analgesia (PCA), a clinical trial developed Patient-Controlled Sleep (PCSL) for chronic intractable insomnia, where the traditional analgesics in PCA were replaced with dexmedetomidine. The duration of PCSL ranged from a few days to four months, and the dose of dexmedetomidine decreased without producing any physical dependency or tolerance symptoms [43]. The sleep quality measured by the Pittsburgh Sleep Quality Index (PSQI) improved immediately after therapy in 12/15 patients, with 7/12 patients achieving consistently improved sleep quality in follow-up. However, it is an off-label use, and the potential side effects of dexmedetomidine with long-term use require further evaluation. A clinical trial was carried out to investigate the effect of intranasal dexmedetomidine on postoperative sleep quality in elderly patients with chronic insomnia while in the hospital after surgery [44]. The Leeds Sleep Evaluation Questionnaire (LSEQ) was used to measure the subjective quality of sleep, and the Acti-graph, PSQI, and Insomnia Severity Index (ISI) were used to measure the objective quality of sleep. This study showed that intranasal administration of dexmedetomidine improves postoperative sleep quality in older patients with chronic insomnia and improves total sleep time, sleep onset latency, and sleep efficiency. Two clinical studies were conducted to evaluate the efficacy and safety of intranasal dexmedetomidine in treating preoperative anxiety-related insomnia by comparing it with lorazepam or placebo [45, 46]. Results showed that intranasal dexmedetomidine could shorten the sleep onset latency, increase total sleep time, and lower ISI.

Xu *et al*. carried out a clinical trial to find out if propofol anesthesia is a useful treatment for individuals with refractory chronic insomnia [47]. Participants in this trial were given a continuous intravenous infusion of 3.0 g/l propofol for two hours for five nights in a row. Subjective sleep assessment was done using the Leeds Sleep Evaluation Questionnaire, while objective analysis of sleep architecture and patterns was done using polysomnography (PSG). All patients with refractory chronic insomnia experienced improvements in both subjective and objective sleep quality after receiving propofol anesthesia. After the therapy, this improvement happened right away and lasted for six months. Neither during the medication administration period nor six months following therapy were any significant adverse effects seen.

### 5.2 Obstructive Sleep Apnea

The use of anesthetics in individuals with obstructive sleep apnea (OSA) was examined in eight studies. Under-diagnosis of OSA is common because of the demanding and time-consuming nature of PSG. Wu *et al.* conducted a clinical trial to evaluate the utility of a short daytime dexmedetomidine-induced PSG for diagnosis of OSA in adults [48]. The drug-induced PSG (DIPSG) was induced by continuous intravenous dexmedetomidine infusion. Sedation depth was monitored and maintained using the Narcotrend index between 50 and 70. This study included 47 OSAS and 39 healthy volunteers and found that sensitivity and specificity for detection of OSA by DIPSG were 92% and 79%, respectively.

Making decisions about surgical treatment for patients with obstructive sleep apnea-hypopnea syndrome requires an understanding of the locations of pharyngeal collapse. During sedative-induced sleep, the upper airway can be directly observed with drug-induced sleep endoscopy (DISE). Rabelo *et al*. conducted a prospective cross-sectional study to investigate the effect of propofol on sleep parameters during DISE [49]. Thirty non-obese participants (6 controls and 24 OSAS patients) received two daytime PSGs, one with DISE and one without DISE. Propofol was continuously infused using a target-controlled infusion pump during the DISE examination. Snoring, apnea-hypopnea index (AHI), oxyhemoglobin saturation (SaO_2_), and sleep macro-architecture were all assessed. The main respiratory parameters, AHI and mean SaO_2_, showed no statistical difference between the two tests, with and without propofol.

However, the minimum SaO_2_ was significantly lower during propofol infusion. Regarding sleep macro architecture, the tests with propofol significantly increased N3 sleep and totally extinguished REM sleep. Propofol significantly changes sleep macro architecture. Heiser *et al*. carried out a study to determine how the various propofol regimes affect the DISE findings and the conclusions and treatment recommendations [50]. Forty-three patients with OSA underwent a DISE procedure using propofol TCI. Three levels of sedation were defined, depending on entropy levels and assessment of sedation: light sedation, medium sedation, and deep sedation. During DISE with propofol TCI, changes in upper airway collapse take place at medium sedation levels. Increasing sedation did not result in changes in the treatment decision. Compared to conventional DISE, two clinical trials used a target-controlled infusion (TCI) sleep endoscopy (DISE-TCI) to mimic snoring and apnea patterns related to a spontaneous sleep situation [51, 52]. The key endpoint is the observation of apneic events and their association with patterns of pharyngeal collapse. Due to its improved accuracy, stability, and safety, the DISE-TCI approach was recommended as the preferred method for performing sleep endoscopy in both trials.

Three studies were conducted to compare the effects of propofol and dexmedetomidine on the upper airway collapse pattern and cardiopulmonary parameters of patients with obstructive sleep apnea undergoing DISE. Yoon *et al.* discovered that though dexmedetomidine caused slightly less upper airway narrowing than propofol, both medications consistently resulted in partial or total obstruction in all locations [53]. Compared to propofol, dexmedetomidine offered less respiratory depression and more hemodynamic stability. Results from Viana *et al.* were also consistent with these findings [54].

### 5.3 Perioperative Sleep Disorders

Thirty-one clinical trials were carried out to investigate the potential therapeutic benefits of anesthetics for perioperative sleep disturbances. Of them, 20 studies investigated the effect of dexmedetomidine on perioperative sleep, 6 investigated the use of propofol, and 5 investigated the use of stellate ganglion block.

Dexmedetomidine, a potent α-2-adrenergic agonist, is widely used as a sedative in critically ill patients. Three trials were intended to examine the effect of dexmedetomidine administration on sleep quality in nonmechanically ventilated ICU after surgery [55-57]. The dose of dexmedetomidine ranged from 0.1 μg·kg^-1^·h^-1^ to 0.6 μg·kg^-1^·h^-1^. According to the findings of these trials, dexmedetomidine is a safe and clinically effective sedative that can be used to improve sleep efficiency and prolong total sleep duration in critically ill patients in the intensive care unit who are not on mechanical ventilation. Two of them also discovered the application of dexmedetomidine could raise the percentage of stage N2 by using PSG to identify changes in sleep architecture during DEX use. However, a clinical investigation of ICU patients on mechanical ventilation revealed that, despite some patterns of improvement, low-dose dexmedetomidine infusion did not significantly enhance the sleep quality pattern [58]. Postoperative sleep disorder is common and may cause aggravated postoperative pain, delirium, and poor prognosis. Numerous investigations have been carried out to examine how the administration of dexmedetomidine during surgery affects the quality of sleep following surgery [59-63]. Even though dexmedetomidine used during surgery has been demonstrated to improve the quality of postoperative sleep for surgical patients significantly, these trials differed greatly from one another. First, there were significant distinctions in the surgeries that individuals underwent, ranging from open surgery to radical mastectomy. Second, there were notable differences in the timing, dosage, and route of administration of dexmedetomidine. The route of dexmedetomidine includes intravenous, oral, nasal packing, nasal spray, *etc*. The dosage of dexmedetomidine changed from 0.02 μg.kg^-1^.h^-1^ to 0.5 μg.kg^-1^.h^-1^. Both intraoperative and postoperative administration of dexmedetomidine had been detected. Third, there were numerous subjective and objective methods for assessing the quality of sleep. The Pittsburgh Sleep Quality Index (PSQI) and the Subjective Sleep Quality Value (SSQV) are examples of subjective metrics; Polysomnography is the objective method.

The effects of total intravenous anesthesia with propofol on the quality of postoperative sleep were investigated in two studies, with conflicting findings [64, 65]. While Ding *et al.* [64] discovered that total intravenous anesthesia based on propofol had minimal impact on the cognitive function and quality of sleep of elderly patients following surgery, Gastrointestinal Endoscopy (GE) with propofol sedation had a negative impact on sleep quality for seven days following GE and not for three weeks. Two studies were carried out to examine the effects of propofol-based total intravenous anesthesia and sevoflurane-based inhalation anesthesia on the quality of postoperative sleep in patients undergoing laparoscopic surgery or total laparoscopic hysterectomy [66, 67]. Niu *et al.* demonstrated that intravenous and inhalation maintenance anesthesia had similar postoperative PSQI values. In contrast to patients in the sevoflurane group, Li *et al.* discovered that the degree of postoperative sleep efficiency was higher on Sleep POD1 and Sleep POD3 when the procedure was carried out under propofol anesthesia. Ultrasound-guided Stellate Ganglion Block (SGB) has been shown in five clinical trials to reduce postoperative sleep disturbances in patients undergoing various surgical procedures [9, 66-71].

## 6 APPLICATION OF ANESTHESIA IN SLEEP DISORDERS (TABLE 1)

### 6.1 Dexmedetomidine

#### 6.1.1 Sedation and Arousal Mechanisms of Dexmedetomidine

It had been speculated that the sedative mechanism of dexmedetomidine resulted from its interaction with α2 adrenergic receptors on the presynaptic membrane of norepinephrine neurons in the locus coeruleus, reducing the release of the excitatory neurotransmitter norepinephrine through the G protein-coupled receptor [3]. However, current evidence does not support this view. Hu *et al*. demonstrated that even in mice devoid of norepinephrine synthesis, dexmedetomidine could induce loss of consciousness [72]. Zhang *et al*. [73] selectively knocked out α2 adrenergic receptors in the LC of mice and discovered that low dose dexmedetomidine (<100 μg/kg) can induce sedation, while high doses (>400 μg/kg) cannot induce loss of consciousness. These results suggest that the sedative and unconsciousness effects of dexmedetomidine are caused by separate neural circuits. Low-dose dexmedetomidine-induced sedation is independent of α2 adrenergic receptors in the LC. Lu *et al.* [6] showed that the addition of yohimbine (selective α2 adrenergic receptor antagonist) to mouse pituitary GH3 cells could not reduce the inhibitory effect of dexmedetomidine on hyperpolarized cation current, suggesting that dexmedetomidine may reduce the excitability of nerve cell membranes by directly blocking the ionic current. The sedation mechanism of dexmedetomidine is still controversial and needs further study.

**Fig. (2):**
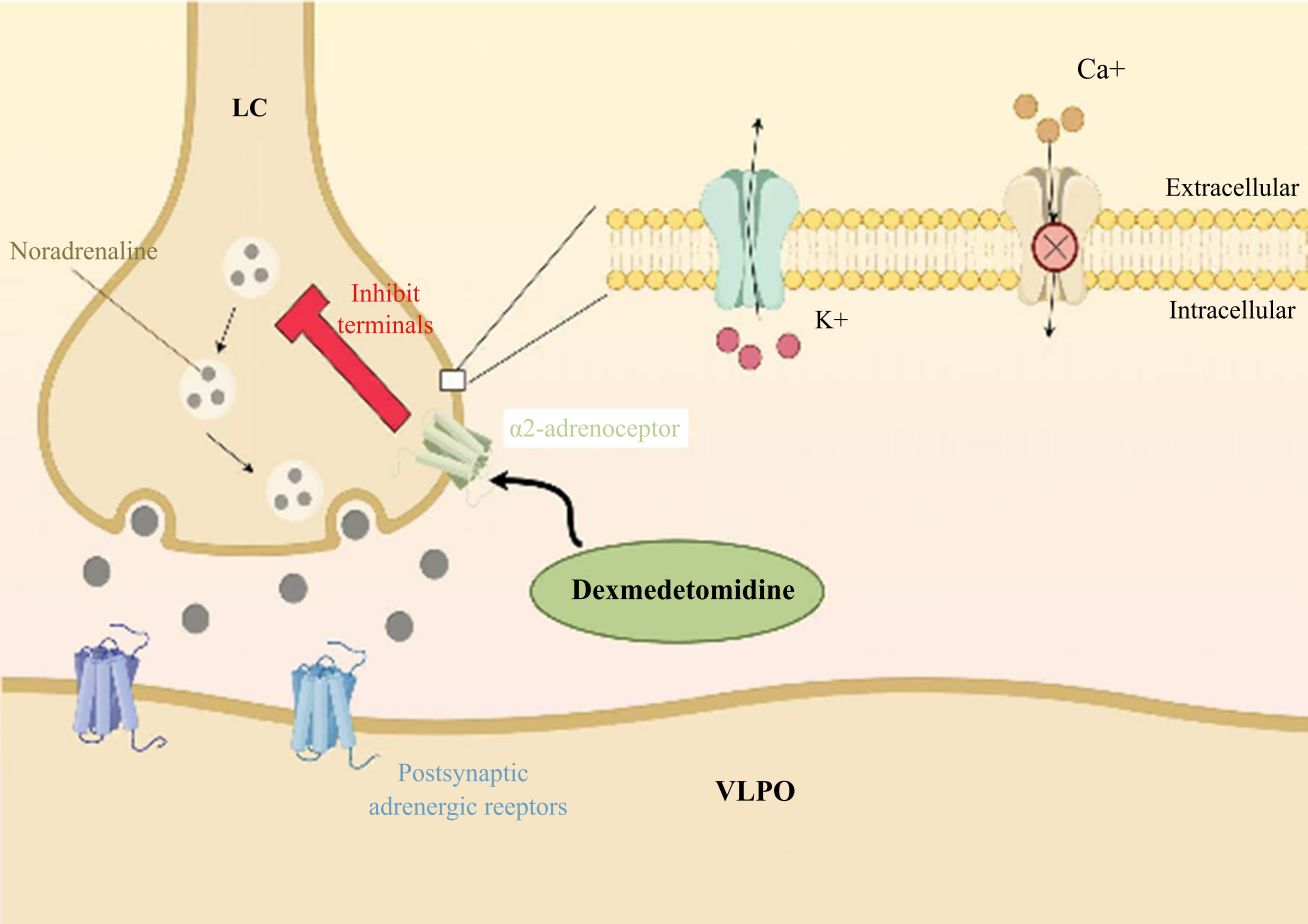
Simplified mechanism of dexmedetomidine at the presynaptic membrane. After dexmedetomidine binds to α2-adrenoceptors in the Locus Coeruleus, transmembrane signaling results in activation of an inwardly rectifying potassium channel facilitating a K^+^ efflux and inhibition of voltage-gated Ca^2+^ channels. The resulting hyperpolarization decreases the firing rate of Locus Coeruleus neurons and allows presynaptic inhibition of their terminals.

The most significant difference between dexmedetomidine and other sedatives is its ability to maintain arousal ability while causing the removal of consciousness. Guldenmund *et al*. [74] conducted brain resting-state functional magnetic resonance imaging on volunteers and revealed that the thalamic functional connectivity with the medial prefrontal/anterior cingulate cortex and Mesopontine were reduced the least during dexmedetomidine-induced unresponsiveness and the most during propofol-induced unresponsiveness. The reduction in N3 sleep was intermediate between that of dexmedetomidine and propofol. These network effects may explain the quick recovery of directed reactivity to external stimuli during dexmedetomidine sedation. Reanimation during general anesthesia can be induced by electrically stimulating VTA or by activating dopaminergic neurons in this region, which is a crucial component of the ascending arousal pathway [75, 76]. According to Qiu *et al*. [77], dexmedetomidine administrated intraperitoneally stimulated dopamine neurons in the ventral tegmental region and raised dopamine levels in the associated forebrain projection areas (Fig. **2**).

#### 6.1.2 Application of Dexmedetomidine in Sleep Disorders

##### 6.1.2.1 Chronic Insomnia

Insomnia is characterized by limited sleep duration, difficulty falling and/or staying asleep, or poor quality sleep that affects daytime performance even when there is enough opportunity for sleep [14]. In contrast to zolpidem, dexmedetomidine was demonstrated by Akeju *et al.* [30] to produce biomimetic sleep, increase stage N3 sleep, and not affect the psychomotor alertness test. This showed that it was possible to induce biomimetic N3 sleep pharmacologically. Additionally, they suggested that alpha-2 adrenergic agonists might be a novel class of drugs that improve sleep while simultaneously having neurocognitive sparing advantages. An *et al.* [43] applied the Patient-Controlled Sleep method to 15 patients with chronic insomnia, enabling them to administer quantifiable doses of dexmedetomidine as needed at night for sedative therapy. They discovered that 12 patients experienced an immediate improvement in their subjective sleep assessment following therapy, and 7/12 patients experienced consistently improved sleep quality during follow-up without experiencing any significant adverse events. The long-term effect of dexmedetomidine on chronic insomnia was preliminarily examined.

Chronic insomnia may be treated with dexmedetomidine. The present research is still in the early stages of investigation, and more extensive clinical trials should be carried out to offer stronger proof of its effectiveness.

##### 6.1.2.2 Obstructive Sleep Apnea

Obstructive Sleep Apnea (OSA) is the most prevalent sleep-disordered breathing in the general population, which is characterized by complete or partial intermittent obstruction of the upper respiratory tract during sleep [78].

Polysomnography (PSG) is the gold standard for the diagnosis of OSA, but due to the demanding and time-consuming nature of PSG, its clinical application is limited. Several research has investigated whether shortening the diagnostic period of conventional PSG can improve its ability to diagnose OSA. Dexmedetomidine was taken to induce a short daytime PSG for the diagnosis of OSA because of its benefits, which include minimal effects on posture and sleep architecture, insignificant respiratory depression, and an arousable feature [74, 79, 80]. Xu *et al.* [81] discovered a greater apnea-hypopnea index (AHI) and a lower minimum oxygen saturation after dexmedetomidine sedation, as well as the absence of REM sleep. Wu *et al*. [48] found that dexmedetomidine-induced PSG had substantially higher minimal oxygen saturation and sleep efficiency than conventional PSG. There were no significant differences in other respiratory events between the conventional and dexmedetomidine-induced PSG. The sensitivity and specificity for detecting OSA with dexmedetomidine-induced PSG were 95% and 85%, respectively. These inconsistent findings may be attributed to the fact that the mean dexmedetomidine dosage and induced sleep duration differed between the two groups. A study by Xu *et al*. used a substantially greater mean dose of dexmedetomidine for inducing sleep (104.60 ± 27.93 μg) than Wu *et al*.'s (56.89 ± 9.88 μg). Sedation depth was commonly assessed using EEG of PSG, the occurrence of respiratory events, and snoring. Crucially, over-sedation is a risk when a high dose of dexmedetomidine is administered without an objective assessment of the sedation level. Furthermore, as the REM stage happens every 90 to 120 minutes [48], it is difficult to record REM sleep with a short sleep duration (20.29 ± 11.64 min). Wu *et al.* extended the sleep duration to 90 and 100 minutes in OSA patients and were the first to reproduce a complete sleep cycle during dexmedetomidine-induced sleep [48]. To verify if dexmedetomidine-induced PSG is a useful screening method for OSA and other sleep-disordered breathing detection, more studies utilizing longer durations of induced sleep and objective sedation evaluation are needed.

Despite the growing use of DISE, the best medicine for resembling physiological sleep remains unknown. Compared to propofol and midazolam, dexmedetomidine is recommended for DISE due to its minimal respiratory effects, more stable cardiopulmonary profile, and capacity to trigger natural sleep loops [82-84]. However, propofol and midazolam exhibit a greater extent of tongue base collapse and lower oxygen levels, while the oxygen nadir during propofol sedation is most similar to that during normal sleep [54]. Despite the fact that most research supports the use of dexmedetomidine for DISE because of its stable respiratory profile, there is insufficient evidence comparing the degree of obstruction with EEG, simultaneously analyzing the stages of sleep induced by dexmedetomidine or propofol. Patients typically suffer the highest level of upper airway obstruction after entering REM sleep [85]. Therefore, the one that would most closely resemble the degree of obstruction observed in REM appears to be the more suitable one for DISE. Wu *et al*. [48] found that 30% of the OSA patients who were sedated with dexmedetomidine observed REM sleep during the 90-minute sleep interval. Since the average DISE time was 139 minutes, it's probable that patients who were sedated with dexmedetomidine naturally transitioned into REM sleep within this period. While developing a perfect sedative is impractical, improving our knowledge of the benefits and drawbacks of different drugs could lead to more effective clinical applications.

##### 6.1.2.3 Perioperative Sleep Disorders

Sleep disorders are common among surgical patients, with 8.8% to 79.1% of patients suffering from sleep disorders before surgery [86]. Sleep disorders may be triggered or aggravated after surgery due to circadian rhythm disturbance, pain, and opioid use. 49.7% of patients may suffer from sleep disorders after surgery for more than 1 year [87]. Perioperative sleep disorders can prolong the recovery time of surgical patients and increase the incidence of postoperative complications and mortality [88].

Dexmedetomidine has been shown in several trials to have a positive effect on sleep quality in patients in the intensive care unit (ICU) or following surgery, as measured by objective tools or sleep questionnaires [61, 89-92]. Normal sleep patterns and cycles are essential for maintaining normal physiological and mental processes. Even in certain fast-track surgeries involving regional anesthesia [93], patients experienced severe disturbances in their normal sleep patterns, which are characterized by a marked decrease in the amount of slow wave and REM sleep immediately following surgery and a tendency for a rebound phenomenon within the first week following surgery [94]. At the same time, stage N1 sleep was significantly longer [95, 96]. Compared to gamma-aminobutyric acid (GABA) agonists, dexmedetomidine more closely resembles natural NREM sleep [3, 97]. Electroencephalogram (EEG) activity during dexmedetomidine-induced sleep includes spindle and slow-delta oscillations, which are similar to physiological stage N2 sleep [97]. Dexmedetomidine suppresses norepinephrine release by binding to receptors in the locus coeruleus. This results in GABA output from the ventrolateral preoptic nucleus, which in turn induces NREM sleep patterns [89]. Norepinephrine plays a permissive function during REM sleep [98]; hence, inhibiting its release with dexmedetomidine may make REM sleep difficult to obtain. Perioperative administration of dexmedetomidine improves postoperative sleep quality and sleep architecture by shortening stage N1 sleep and prolonging stage N2 sleep while having no significant effect on REM sleep [86].

The effects of dexmedetomidine on postoperative sleep quality were correlated with the dosage and timing of administration. A large sample of patients undergoing nine different types of non-cardiac major surgery was included in a real-world clinical cohort, which showed that low-dose dexmedetomidine had a greater impact on preventing postoperative sleep disturbances than medium- and high-dose dexmedetomidine [99]. Jiang *et al.* [100] compared the effects of oxycodone and dexmedetomidine combinations on postoperative sleep quality and found that larger doses of dexmedetomidine did not further improve sleep but raised the risk of hypotension. Other characteristics such as surgical type, age, prior comorbidity, clinical settings, and medical interventions were also closely associated with the incidence of postoperative sleep disorders [100-102]. Individual variability should be taken into account while investigating the best dexmedetomidine dosage regimen for reducing postoperative sleep problems. Compared to administering dexmedetomidine during the nighttime operation, Song *et al.* discovered that taking it during the daytime operation could enhance subjective sleep quality and postoperative sleep efficiency more [103]. Given the pharmacologic sensitivity influenced by chronobiology and the time-dependent fluctuations in pain, the intraoperative dose of dexmedetomidine and the postoperative analgesic dose for the nighttime operation should be properly raised in comparison to those for the daytime operation [103]. However, Tan *et al.* [104] reported that with the deeper sedative state provided by dexmedetomidine in the daytime, elderly male patients under spinal anesthesia suffered worse sleep on the night of surgery. Similar results were observed in the ICU settings [89]. The optimal dosage of dexmedetomidine and the timing of administration to better improve postoperative sleep disorders will need further study.

Currently, most studies focus on intraoperative or postoperative sleep disorders, while preoperative sleep disorders are rarely paid attention to. It is well known that preoperative stress will increase the incidence of perioperative complications. Further research is required to determine whether preoperative treatment for sleep disturbances might reduce stress and thus provide clinical benefits.

##### 6.1.2.4 Restless Leg Syndrome

The neurological condition known as Restless Leg Syndrome (RLS) is characterized by creeping, nonpainful urges to move lower extremities and is relieved with movements of the legs. RLS has had a low diagnostic rate because there is no “gold standard” for the condition. According to Ohshiea *et al.* [105], after receiving an intravenous infusion of 0.2 μg·kg^-1^·h^-1^ dexmedetomidine, the patient's RLS symptoms worsened and spread to her upper extremities. Upon increasing the infusion of dexmedetomidine from 0.2 to 0.4 μg·kg-^1^·h^-1^, the patient experienced a three-hour sleep, and nearly all symptoms were alleviated. In this instance, a suspected exacerbation of RLS was effectively managed with the aid of a dexmedetomidine infusion.

#### 6.1.3 Addiction and Withdrawal Symptoms with Dexmedetomidine

As dexmedetomidine’s clinical use continues to grow, the situations of long-term and high-dose use are gradually increasing, and the issue of addiction and withdrawal has started to receive attention from researchers.

There is little research on dexmedetomidine addiction, and most of it is based on animal studies. According to a 2016 study by Uskur *et al*., in a rat-conditioned place preference model, dexmedetomidine demonstrated neurotoxic effects similar to those of morphine, indicating that it might have potential addictive properties [106]. Nevertheless, Uskur’s most recent work showed that propofol, but not dexmedetomidine, significantly increased locomotor sensitization through the central nitrergic system [107]. Dexmedetomidine may have a less psychostimulant-type addictive potential than propofol.

It has been confirmed that sudden discontinuation of dexmedetomidine after prolonged use might result in withdrawal symptoms in critically ill pediatric patients [108, 109]. These symptoms, which mainly manifest as tachycardia, hypertension, and agitation, occur in approximately 35% of cases [110]. The cumulative dose of dexmedetomidine greater than 60 ug/kg is an independent risk factor, and its mechanism may be the hemodynamic changes caused by norepinephrine rebound. The prevalence of dexmedetomidine withdrawal in adult patients ranged from 20% to 64%, with significant variation across trials [111]. Following extended sedation with dexmedetomidine, 30.3% of the 165 critically ill adult patients in the Pathan *et al.* research experienced withdrawal symptoms, which is comparable to the rate seen in children [111]. The median dose of dexmedetomidine was 0.56 ug, and the median administration time was 52.5 hours. 2225 ug was the median cumulative dose. The study also discovered that when compared to abrupt discontinuation, weaning did not lower the frequency of withdrawal symptoms. Salah *et al.* [112] demonstrated that in critically ill adult patients, a prior medical history of hypertension constituted a separate risk factor for dexmedetomidine withdrawal syndrome.

### 6.2 Propofol

#### 6.2.1 Sleep Improvement Mechanisms of Propofol

Propofol is a commonly used, fast-acting anesthetic that does not accumulate in the body. The mechanism of propofol's anesthetic activity is linked to GABA-induced suppression of the post-synaptic potential. In sleep-deprived rats, propofol treatment resulted in almost no sleep rebound, a faster rate of recovery to the control level, and an increase in the NREM sleep delta power [113]. These findings suggest that some of the sleep debt was made up for during propofol anesthesia. Recovery from REM and NREM sleep is facilitated by propofol anesthesia [26].

Boveroux *et al.* [114] used blood oxygen level-dependent functional magnetic resonance imaging on healthy humans in propofol-induced sedation and awareness states to identify the mechanism by which propofol improves sleep quality. According to their research, functional connectivity in specific thalamocortical and higher association corticocortical networks is inversely correlated with greater propofol dosages. Additionally, Murphy *et al.* [115] examined the cerebral responses to propofol anesthesia using high-density electroencephalography. They discovered that reduced consciousness is linked to slow waves in propofol anesthesia. Namely, propofol anesthesia seems to induce a sleep-like state [116]. Biochemical analysis also confirmed improved sleep quality following propofol anesthesia. Following sleep deprivation, there was a reduction in excitatory and inhibitory neuronal function in the cortices and thalamus and a disruption in the equilibrium of Glu/Gln metabolism. Glutamate metabolic kinetics were selectively restored by propofol in several brain regions, including the striatum, thalamus, frontal, temporal, and parietal cortex [117]. Anesthesia with propofol was also found to be more effective in restoring extracellular Glu and GABA to normal in the CA1 area of the hippocampus of rats that had been sleep-deprived for 24 hours [118]. A precise balance between glutamatergic and GABAergic systems is essential for restoring the best brain function. Effective glucose metabolism and regional functional integrity and connections are essential for brain health [119, 120], and these are impacted by sleep deprivation [121]. Propofol medication, but not sevoflurane treatment, reversed the considerable reduction in global glucose metabolism and functional connectivity in the vast majority of brain areas caused by a 4-week sleep deprivation [117].

There have also been reports of neuroprotective benefits from propofol. According to Liu *et al.* [113], propofol exerts neuroprotective benefits by reducing neuronal inflammation and changing the microglia phenotype from an M1 to an M2-activated state, which could reduce SD-induced cognitive impairment and disturbance of the circadian rhythm. Dai *et al.* [122] demonstrated that by preventing excessive autophagy and mitophagy in hippocampus neurons, propofol anesthesia could mitigate learning and memory deficits in sleep-deprived rats. Additionally, earlier studies indicated that DAT in the VTA area might be connected to the improvement in sleep and anxiety levels in sleep-deprived rats given propofol [123].

#### 6.2.2 Application of Propofol in Sleep Disorders

##### 6.2.2.1 Chronic Insomnia

According to Tung *et al.* [26], rats who were sleep-deprived and anesthetized with propofol recovered in the same way as rats who were not sleep-deprived, indicating that anesthesia and sleep may have similar regulatory mechanisms. Their findings suggested that patients suffering from refractory chronic primary insomnia might benefit therapeutically from anesthetic drugs. In order to evaluate the safety and effectiveness of propofol in patients with refractory chronic insomnia, Xu *et al.* [47] carried out a preliminary study. They found that a 2-hour continuous intravenous infusion of 3.0 g/L propofol for five nights significantly improved the quality of sleep, as evidenced by the immediate and sustained improvement in both subjective and objective assessments of sleep onset, duration, and quality. Additionally, the 5-day propofol medication gave the patients the confidence they needed to fall asleep. Propofol is an anesthetic that is frequently used, quick to take effect, and does not build up in the body. Propofol’s anesthetic effect mechanism is linked to GABA-induced post-synaptic potential inhibition [26]. Furthermore, propofol has been showed to block the buildup of excitatory amino acids like glutamate and to restore the hippocampal excitatory and inhibitory neurotransmitter release [124]. Propofol has also been shown to have neuroprotective properties [125]. All of these mechanisms may contribute to the positive therapeutic outcomes of propofol in patients with refractory chronic insomnia.

Propofol-induced sleep has been shown by Xu *et al.* [47] to be a safe and effective alternative treatment for individuals with refractory chronic primary insomnia. A multi-center clinical trial should be conducted to corroborate these findings further.

##### 6.2.2.2 Obstructive Sleep Apnea

The importance of DISE has increased due to the growing requirement for an accurate preoperative examination. The effectiveness of this examination in accurately identifying the obstructive sites and improving surgical outcomes has been demonstrated in earlier research [126].

Propofol is frequently used in DISE because of its pharmacological properties, which include a short half-life and minimal side effects. Rabelo *et al.* [49] performed a cross-sectional investigation to identify the areas of obstruction and discovered that propofol DISE, when combined with target-controlled infusion, was a useful medication for endoscopic examination of patients with OSA. The mean propofol concentration reported by Diprifusor was 2.31 ± 0.6 μg/ml, while half of patients lost consciousness at doses below 1.7 μg/ml. Despite the wide range of pharmacodynamics, most patients required a concentration of 1.5 to 2.5 μg/ml to lose consciousness. In DISE, comparatively little propofol is required to produce sleep. Additionally, Hillman *et al.* [127] detected a loss of consciousness at propofol concentrations ranging from 1.5 to 4 μg/ml. Using an electroneuromyogram, they saw a noticeable rise in genioglossus tonus at these concentrations. However, there was an apparent reduction in tonus at greater concentrations. This finding emphasizes it is crucial to avoid over-sedation with propofol in order to prevent excessive muscular relaxation. Heiser *et al.* [50] studied the relationship between the obstruction patterns and the increasing sedation depth induced by propofol during DISE. They discovered that 3.2 μg/ml of medium sedation was adequate to guide subsequent OSA surgical procedures and that further increasing the level of sedation had no effect on the choice of treatment. Two main side effects of conscious sedation during DISE include oversedation and airway obstruction [128]. Propofol's action on the upper airway is dose-dependent, according to several studies [49, 82]. For this reason, it's critical to use the right dosage of propofol during DISE to generate snoring symptoms, with or without obstruction, without inducing respiratory depression.

The infusion pump achieves the same characteristics in the intraindividual examination, allowing for better standardization. When Kezirian *et al.* [129] investigated underexamined patient consistency in 108 DISEs using propofol, they found that there was a moderate agreement for the proportion of obstruction in each region and a high agreement for the obstructive site and the primary structure causing obstruction. Another characteristic of the TCI was the capacity to sustain the most consistent propofol concentration. When De Vito *et al.* [51] contrasted a TCI technique with manual sleep induction, they found that the TCI method was more reliable at sustaining sedation with propofol. Additionally, they noted that no patient in the TCI protocol needed oxygen supplementation, whereas 10% of patients using the manual technique needed it because of a deeper level of sedation.

Propofol significantly alters the structure of sleep. While propofol maintained the proportion of N2 sleep, it altered the distribution of the other sleep stages, boosting N3 sleep, decreasing N1 sleep, and eliminating REM sleep [49]. Since RSD incidents mostly happen during REM sleep in some individuals, REM sleep must be absent following propofol sedation. According to Rabelo *et al.* [49], propofol does not affect the two primary respiratory parameters, AHI and mean SaO_2_, although it considerably alters the architecture of sleep. The alterations to sleep architecture brought on by propofol emphasize the significance of thoroughly investigating the effects of propofol on sleep parameters.

##### 6.2.2.3 Perioperative Sleep Disorders

According to Hu *et al.* [8], patients who had minor gynecologic surgery experienced significantly better sleep quality on the first postoperative night measured by the PSQI when propofol anesthesia was used instead of sevoflurane anesthesia. Ding *et al.* [64] also found that propofol-based total intravenous anesthesia may lower the prevalence of sleep disturbances by stimulating melatonin secretion and blocking the release of cortisol and other inflammatory cytokines. Propofol anesthesia seems to facilitate people's recovery from sleep deprivation and preserve the homeostasis of both REM and NREM sleep [26]. However, this was not the case during or after sevoflurane anesthesia. According to earlier research, propofol used in intensive care units also helped patients' sleep structure return to normal [130, 131]. Conversely, a Cochrane study found little evidence to support the idea that propofol enhances both the amount and quality of sleep in the ICU [7]. Further research is required to determine the cause of the disparity between these studies, which is likely due to the acute *versus* chronic experimental settings, propofol dosage [132], and study individuals' health conditions [123].

Surgical patients often experience chronic sleep difficulties prior to surgery, sleep disturbances following surgery, or both. Postoperative sleep disorders, which are common in postoperative surgical patients and contribute to poor surgical outcomes, can be caused by preoperative comorbidity, anesthesia, surgical trauma, postoperative pain, and anxiety. Adverse cardiovascular events, delayed recovery, and worse cognitive function are all strongly associated with poor sleep quality [93, 133]. In a chronic REM sleep deprivation model in rodents, Zhu *et al*. [117] discovered that propofol, but not sevoflurane, improved the quality of rats' sleep after extended sleep deprivation by enhancing the functional connectivity of the medial prefrontal cortex and the dynamics of brain glutamate and glucose metabolism. Propofol anesthesia can reduce the risk of delayed neurocognitive recovery one week following surgery as compared to sevoflurane anesthesia [134]. Taken together, these studies imply that propofol may be a preferred anesthetic option for patients with sleep disorders who undergo surgery, anesthesia, or both, especially those who are at a high risk of developing sleep disorders following both procedures.

#### 6.2.3 Complications Associated with the Use of Propofol

The adverse effects of propofol are well-documented, with the most common being pain on injection. Other side effects include cardiovascular events, including bradycardia and hypotension, as well as metabolic events like hyperlipidemia caused by lipid formulation infusion.

Injection pain is widespread in adults, ranging from 28% to 90% [135, 136]. This pain is caused by the composition of propofol, which is an oil-in-water emulsion. This formulation was employed because of the limited solubility of propofol in aqueous solution. Remarkably, almost one-third of patients say that receiving propofol injections causes them to suffer from severe or excruciating pain.

The effects of propofol on the cardiovascular system are wide. The most noticeable effect is the reduction in systemic blood pressure combined with a decrease in cardiac output. This impact is dose-dependent and even occurs at sedative doses. It is more pronounced in elderly and physiologically compromised patients [137]. Vascular resistance and sympathetic tone both significantly decrease, which at least partially mediates the impact. Additionally, propofol increases cardiovascular depression by suppressing the physiological baroreflex reactions [138].

Propofol is also a potent ventilatory depressant. When used clinically, propofol can easily cause respiratory depression in patients. Patients who receive even an induction dose may experience a decrease in their tidal volume and respiratory rate, as well as apnea, which poses a serious risk to anesthesia. A prospective multicenter investigation conducted in Germany showed that from December 2011 to August 2014, 15 patients (0. 005%) had sedation-related deaths during gastric endoscopic surgery with propofol as the main anesthetic drug. One of the most common causes of death is hypoxia, secondary to respiratory depression. The mechanism of propofol-induced respiratory depression is still poorly understood. It affects central chemoreceptor sensitivity, which decreases ventilatory responses to hypoxia and hypercapnia, causing dose-dependent ventilation interference [139-141]. Apnea is brought on by greater dosages of propofol [142].

In a recent thorough analysis, Uzbay *et al.* assessed the abuse and addiction potential of propofol using data from both clinical and experimental research [143]. Scientific data supported the notion that propofol has a strong potential for abuse and produces significant addiction in both experimental animals and humans. A detailed analysis of case reports, as well as limited clinical, observational, and cross-sectional studies, found an increasing trend in poisonings and deaths related to propofol abuse. Although some countries have implemented strict monitoring and control measures for propofol [144, 145], recent studies have indicated that the problem persists. Uzbay *et al.* stressed the significance of using propofol with extreme caution in medical practice, arguing that it should be included in the list of controlled substances. Due to its narrow therapeutic margin, propofol should only be administered by professionals who are trained and experienced in general anesthesia [143].

### 6.3 Stellate Ganglion Block

Stellate Ganglion Block (SGB) is widely used clinically and is commonly used to treat vegetative disorders [146]. Studies showed that SGB can improve sleep disturbances caused by sympathetic hyperexcitability by inhibiting the sympathoadrenal system and reducing nociceptive transmission [147]. Yan *et al*. [9] performed preoperative ultrasound-guided left-sided SGB in patients undergoing radical gastrointestinal malignancy surgery and found that SGB ameliorates postoperative sleep disorders (POSD), decreases postoperative inflammatory response, maintains perioperative hemodynamic stability, and increases melatonin levels.

The ameliorative effect of SGB on POSD may be attributed to SGB’s ability to reduce the central sympathetic tone and regulate the equilibrium of body systems. By reducing sympathetic excitability, suppressing perioperative stress, and decreasing the secretion of adrenocorticotropin-releasing hormone, norepinephrine, and epinephrine, SGB can regulate cortical function, endocrine system function, and autonomic nervous system function, thus physiologically enabling patients to fall asleep quickly and maintain an optimal sleep state [147]. SGB can enhance sleep quality by inhibiting the sympathetic plexus surrounding the vertebrobasilar and posterior cerebral arteries, thus increasing cerebral blood flow and promoting the recovery of cortical function [148]. The beneficial effect of SGB on POSD may also be attributed to its regulation of pineal melatonin secretion, which initiates the physiological rhythm restoration of melatonin and alleviates sleep disorders and sleep rhythm disturbances caused by fluctuating melatonin levels [149]. Reduced perioperative inflammatory response may also play a role in the ameliorative effect of SGB on POSD [150].

## CONCLUSION

General anesthesia and sleep state have both similarities and differences. Studying the effects of general anesthetics on sleep will help to improve sleep quality, reduce the incidence of sleep disorders, guide clinical medication, and improve the prognosis of patients.

As an intravenous anesthetic, dexmedetomidine has been increasingly applied in other disciplines. Recent studies have demonstrated the application value of dexmedetomidine in the diagnosis and treatment of sleep disorders, and the results are encouraging, indicating that it is a drug with great clinical potential. However, there are also a lot of flaws in the current research, particularly because many studies are still in the preliminary exploration stage, and large randomized clinical trials are required to validate the results in the future. Furthermore, the optimal regimen, which includes the dosage, method of administration, treatment cycle, and interval cycle, needs to be investigated.

Propofol is a commonly used anesthetic drug with a rapid onset of action and does not accumulate in the body. Propofol anesthesia has been found to improve sleep quality after sleep deprivation and promote the recovery of REM and NREM in animal models and small clinical studies. There are limited studies on the effects of propofol on the sleep of patients with sleep deprivation. Furthermore, there is heterogeneity among different studies in terms of research design, methodology, comparator drugs, and disease severity. It is not sufficient to conclude whether the administration of propofol can improve the sleep quality and quantity of patients with sleep disorders. Large-scale randomized controlled clinical studies are required to enhance the certainty of this review. Stellate ganglion block has also been shown to reduce the incidence of postoperative sleep disorders.

A number of side effects, including bradycardia, hypotension, respiratory depression, and hyperlipidemia, can occur when propofol is used in therapeutic settings. The use of propofol has also been proven to have a strong potential for abuse and addiction in both experimental animals and humans. Abrupt withdrawal of dexmedetomidine after long-term use may lead to withdrawal reactions in critically ill children and adults. Therefore, the safety of long-term use of anesthetic drugs for the treatment of sleep disorders still needs to be further observed in future studies.

In conclusion, anesthetic drugs are a potential therapeutic option for the diagnosis and treatment of sleep disorders. However, research in this area is limited, and there is significant heterogeneity among different studies. Large-scale clinical studies are needed to enhance the certainty of this review. Similarly, the safety issues associated with the application of anesthetic drugs in patients with sleep disorders cannot be ignored. The application of aesthetics should be operated and monitored by professional doctors, especially in clinical studies of refractory sleep disorders.

## STUDY LIMITATIONS

The current research on the application of anesthetic agents in the management of sleep disorders presents several limitations that warrant careful consideration. First, the literature on the use of anesthetic drugs for treating sleep disorders is limited, precluding the ability to draw definitive conclusions. While preliminary findings are promising, the lack of large-scale, well-designed randomized controlled trials limits the generalizability and reliability of the results. Second, significant heterogeneity exists across studies in terms of research design, methodologies, comparator drugs, and patient populations (*e.g*., disease severity and comorbidities), which complicates the synthesis of evidence and the establishment of standardized treatment protocols. Third, safety concerns associated with the long-term use of anesthetics, such as bradycardia, hypotension, respiratory depression, hyperlipidemia, and the potential for abuse or withdrawal reactions, have not been adequately addressed, highlighting the need for rigorous safety monitoring in future studies. Fourth, the optimal dosing regimens, administration methods, treatment durations, and interval cycles remain undefined, emphasizing the need for more comprehensive investigations. While anesthetic agents hold potential as therapeutic options for sleep disorders, the current evidence is insufficient to support their routine clinical application. Future research should prioritize large-scale clinical trials, standardized protocols, and long-term safety assessments to address these limitations and enhance the clinical applicability of these agents.

## Figures and Tables

**Table 1:** Summary of anesthesia agents and techniques for the treatment of sleep disorders.

**Treatment**	**Mechanisms**	**Chronic Insomnia**	**Obstructive Sleep Apnea**	**Perioperative Sleep Disorders**	**Additional** **Applications**
Dexmedetomidine	1. α2 adrenoceptor agonists. 2. Preserving the within-network functional connectivity. 3. Activating dopamine neurons in the VTA.	1. Induce biomimetic N3 sleep. 2. Improve the subjective sleep quality of chronic insomnia patients in the short and long term.	1. Induce REM sleep, not restrict the free change of position, and not increase the severity of apnea. 2. The sensitivity and specificity of dexmedetomidine induced sleep detection of OSA were 92% and 79%.	1. Low dose dexmedetomidine can improve postoperative sleep quality, larger doses did not further improve sleep but increased the risk of hypotension. 2. Improved sleep efficacy and subjective sleep quality with the intraoperative use of dexmedetomidine during the nighttime operation, while another showed worse sleep on the night of surgery using dexmedetomidine.	One case reported that increasing the dexmedetomidine infusion from 0.2 to 0.4 μg/kg/h improved the restless leg syndrome symptoms.
Propofol	1. Work on GABA receptors. 2. Promot the restoration of disturbed neurotransmitter release in the hippocampus neuroprotective effects.	Improved quality of sleep after a 2-h continuous intravenous infusion of propofol for five consecutive nights.	Provide a standardized and controlled state of sedation and re-create snoring and apneic pattern in the best possible way to simulate a spontaneous sleep situation.	Propofol anesthesia may effectively reduce the incidence of sleep disorders and improve the sleep quality on the first postoperative night.	-
Stellate ganglion block	1. Reduce sympathetic excitability. 2. Inhibit the sympathetic plexus surrounding the vertebrobasilar and posterior cerebral arteries. 3. Regulate pineal melatonin secretion. 4. Reduce perioperative inflammatory response.	Ameliorate postoperative sleep disorders and maintains perioperative hemodynamic stability.	N/A	N/A	N/A

**Abbreviations**: VTA, Ventral Tegmental Area; OSA, Obstructive sleep apnea.

## LIST OF ABBREVIATIONS

AHI: Apnea-hypopnea Index
BF: Basal Forebrain
EEG: Electroencephalogram
GABA: Gamma-aminobutyric Acid
GE: Gastrointestinal Endoscopy
LC: Locus Coeruleus
OSA: Obstructive Sleep Apnea
PCA: Patient-controlled Analgesia
PSG: Polysomnography
REM: Rapid Eye Movement
RLS: Restless Leg Syndrome
SGB: Stellate Ganglion Block
SCN: Suprachiasmatic Nucleus
TCI: Target-controlled Infusion
TMN: Tuberomammillary Nucleus
VTA: Ventral Tegmental Area
VLPO: Ventrolateral Preoptic Nucleus
